# NFAT inhibitor 11R-VIVIT ameliorates mouse renal fibrosis after ischemia-reperfusion-induced acute kidney injury

**DOI:** 10.1038/s41401-021-00833-y

**Published:** 2021-12-22

**Authors:** Zhi-yong Xie, Wei Dong, Li Zhang, Meng-jie Wang, Zhen-meng Xiao, Yu-hua Zhang, Wan-xin Shi, Ying Huang, Yan Yang, Cui-li Li, Lei Fu, Xing-chen Zhao, Rui-zhao Li, Zhi-lian Li, Yuan-han Chen, Zhi-ming Ye, Shuang-xin Liu, Zheng Dong, Xin-ling Liang

**Affiliations:** 1 The Second School of Clinical Medicine, Southern Medical University, Guangzhou, 510515 China; 2 Division of Nephrology, Guangdong Provincial People’s Hospital, Guangdong Academy of Medical Sciences, Guangzhou, 510080 China; 3 School of Medicine, South China University of Technology, Guangzhou, 510006 China; 4 Department of Cellular Biology and Anatomy, Medical College of Georgia at Augusta University, Augusta, GA USA; 5 Department of Medical Research, Charlie Norwood Veterans Affairs Medical Center, Augusta, GA USA

**Keywords:** acute kidney injury, chronic kidney disease, NFAT2, 11R-VIVIT, renal fibrosis, apoptosis, renal tubular epithelial cells, HK-2 cells

## Abstract

Acute kidney injury (AKI) with maladaptive tubular repair leads to renal fibrosis and progresses to chronic kidney disease (CKD). At present, there is no curative drug to interrupt AKI-to-CKD progression. The nuclear factor of the activated T cell (NFAT) family was initially identified as a transcription factor expressed in most immune cells and involved in the transcription of cytokine genes and other genes critical for the immune response. NFAT2 is also expressed in renal tubular epithelial cells (RTECs) and podocytes and plays an important regulatory role in the kidney. In this study, we investigated the renoprotective effect of 11R-VIVIT, a peptide inhibitor of NFAT, on renal fibrosis in the AKI-to-CKD transition and the underlying mechanisms. We first examined human renal biopsy tissues and found that the expression of NFAT2 was significantly increased in RTECs in patients with severe renal fibrosis. We then established a mouse model of AKI-to-CKD transition using bilateral ischemia-reperfusion injury (Bi-IRI). The mice were treated with 11R-VIVIT (5 mg/kg, i.p.) on Days 1, 3, 10, 17 and 24 after Bi-IRI. We showed that the expression of NFAT2 was markedly increased in RTECs in the AKI-to-CKD transition. 11R-VIVIT administration significantly inhibited the nuclear translocation of NFAT2 in RTECs, decreased the levels of serum creatinine and blood urea nitrogen, and attenuated renal tubulointerstitial fibrosis but had no toxic side effects on the heart and liver. In addition, we showed that 11R-VIVIT administration alleviated RTEC apoptosis after Bi-IRI. Consistently, preapplication of 11R-VIVIT (100 nM) and transfection with NFAT2-targeted siRNA markedly suppressed TGFβ-induced HK-2 cell apoptosis in vitro. In conclusion, 11R-VIVIT administration inhibits IRI-induced NFAT2 activation and prevents AKI-to-CKD progression. Inhibiting NFAT2 may be a promising new therapeutic strategy for preventing renal fibrosis after IR-AKI.

## Introduction

Acute kidney injury (AKI) is a common clinical syndrome and is associated with significant morbidity and mortality. Although the majority of patients may recover from AKI, 29.4% of AKI patients progress to chronic kidney injury (CKD) stage 3 or higher within the subsequent one year [1]. Individuals with AKI have a 4.81-fold increased risk of end-stage kidney disease (ESKD), resulting in a substantial economic burden on public health [2].

Progressive renal fibrosis is the final common and devastating pathway for kidney diseases, including the AKI-to-CKD transition, which is characterized by increased extracellular matrix components and inflammatory cell infiltration [3]. The AKI-to-CKD transition involves multiple signalling pathways and pathogenesis and has become an intervention target for clinical transformation and treatment [4, 5]. Currently, no effective clinical therapy can reverse renal fibrosis and reduce AKI-to-CKD progression. Injured renal tubular epithelial cells (RTECs) are the primary drivers of renal fibrosis, and the maladaptive repair of injured RTECs after AKI leads to residual abnormalities in kidney structure and function [6]. RTEC apoptosis could result in the occurrence and progression of renal fibrosis by generating proinflammatory and profibrotic paracrine factors [7]. Excessive apoptotic RTECs can activate macrophages and fibroblasts, which release various cytokines and accelerate tubular atrophy and renal interstitial fibrosis, eventually leading to CKD [7, 8]. Thus, inhibiting RTEC apoptosis might be a potential therapeutic strategy for the AKI-to-CKD transition [9, 10].

The nuclear factor of the activated T cell (NFAT) family was initially identified as a transcription factor and includes five members. Four of these proteins (NFAT1, NFAT2, NFAT3, NFAT4) are regulated by calcium signalling, while NFAT5 is regulated by osmotic stress [11]. The Ca^2+^-activated phosphatase calcineurin (CaN) dephosphorylates NFATs, promoting NFAT nuclear translocation and activation. NFAT family members are expressed in most immune system cells and are involved in the transcription of cytokine genes and other genes that are critical for the immune response [12]. Moreover, previous work has shown that NFAT2 could also play an important regulatory role in the kidney and is expressed in nonimmune cells, including RTECs and podocytes [13–16]. Recently, substantial evidence suggests that NFAT2 is involved in the apoptosis of several cell types [17, 18]. Our previous studies demonstrated that NFAT2 mediates high-glucose-induced podocytes apoptosis [15, 16]. However, whether NFAT2 is involved in RTEC apoptosis during renal fibrosis has not been investigated. Moreover, previous studies have suggested that NFAT2 promotes the fibrotic process in several organs [19, 20]. To date, no studies have investigated the effects of NFAT2 on renal fibrosis in AKI-to-CKD progression.

11R-VIVIT is a cell-permeable peptide inhibitor of NFAT that can directly inhibit the dephosphorylation of NFAT without affecting the activity of CaN phosphatase or disrupting other CaN-dependent pathways [21, 22]. Moreover, the adverse effects observed in response to traditional CaN inhibitors (CNIs), cyclosporine, and tacrolimus, including the progressive loss of renal function, hypertension, neurotoxicity, and an increased risk of malignancy, have not been observed in response to 11R-VIVIT [21]. At present, 11R-VIVIT has been shown to attenuate albuminuria and alleviate podocyte injury in diabetic kidney disease [15, 16]. However, the effect of 11R-VIVIT on renal fibrosis in the AKI-to-CKD transition has not been investigated. In the current study, we investigated whether 11R-VIVIT has renoprotective effects on subsequent renal fibrosis after ischemia-reperfusion-induced acute kidney injury (IR-AKI).

## Materials and methods

### Patients

Renal biopsy tissues from nine patients with IgA nephropathy (IgAN) accompanied by severe renal fibrosis (*n* = 3), patients with IgAN with mild renal fibrosis (*n* = 3) and adjacent normal tissues from renal cell carcinoma patients (*n* = 3) were obtained after the patients signed written informed consent. Severe renal fibrosis was defined as >25% tubular atrophy/interstitial fibrosis, while mild renal fibrosis was defined as <25% tubular atrophy/interstitial fibrosis. The clinical parameters of the nine patients are provided in Supplementary Table 1. The expression of NFAT1, NFAT2, NFAT3, and NFAT4 was evaluated using the immunofluorescence staining of frozen sections. The study complied with the Declaration of Helsinki and was approved by the Ethics Committee of Guangdong Provincial People’s Hospital (No. GDREC2020199A).

### Animal experimental protocol

Six- to eight-week-old male C57BL/6 mice (weight, 20–25 g) were purchased from the Nanjing Biomedical Research Institute of Nanjing University and were housed at the Animal Centre of Guangzhou Forevergen Biosciences Co., Ltd., which was maintained at a temperature of 23 ± 2 °C and a humidity of 55% ± 5%. All mice were administered a standard diet and water and were housed in a pathogen-free environment and a 12/12 h light/dark cycle. The animal study was approved by the Animal Ethics and Welfare Committee (No. IACUC-G16034). After a two-week adaptation period, the mice were randomly divided into three groups: sham-operated mice (sham, *n* = 18, sham operation was performed without clamping the renal pedicles); ischemia-reperfusion mice (IRI, *n* = 18, mice were subjected to bilateral renal pedicle clamping for 25 min); and ischemia-reperfusion mice that were intraperitoneally injected with 11R-VIVIT (IRI + 11R-VIVIT, *n* = 18, mice were subjected to 25 min of ischemia and received 5 mg/kg 11R-VIVIT on Days 1, 3, 10, 17, and 24 after the operation). Mice were anaesthetized with an intraperitoneal injection of 60 mg/kg 1% pentobarbital sodium. Bilateral ischemia-reperfusion injury (Bi-IRI) was induced by the placement of an atraumatic microaneurysm clamp (RS-5420, Roboz Surgical Instrument Co., Inc., Gaithersburg, MD, USA) on the renal pedicle for 25 min while the mice were warmed with a heating pad (37 °C) throughout the surgery. After surgery, 1 mL of warm saline was intraperitoneally injected for volume substitution. Fifty-four mice in the sham-operated, IRI and IRI + 11R-VIVIT groups were euthanized on the 2nd day (*n* = 18), 14th day (*n* = 18), and 28th day (*n* = 18) after the operation to examine serum creatinine (Scr), blood urea nitrogen (BUN), and renal pathology. Blood samples were obtained from the retro-orbital venous plexus, and the left kidneys were harvested and stored in liquid nitrogen until use. The right kidney, heart, and liver were fixed in 4% paraformaldehyde overnight and embedded in paraffin. Kidney sections (3 μm) were stained with Masson’s trichrome to evaluate renal fibrosis. Heart and liver sections (3 μm) were stained with haematoxylin-eosin (HE) to observe the pathological changes.

### Evaluation of renal function and liver function

Renal function was assessed by measuring Scr and BUN. Scr was determined using the QuantiChrom™ Creatinine Assay Kit (Cat. No. DICA-500, BioAssay Systems, Hayward, CA, USA), and BUN was assessed using the QuantiChrom™ Urea Assay Kit (Cat. No. DIUR-100, BioAssay Systems, Hayward, CA, USA) according to the manufacturer’s protocols. Serum aspartate aminotransferase (AST) was detected using the Cloud-Clone Corp Kit (Lot. L210820841, Cloud-Clone Corp, TX, USA).

### Cell culture and treatment

HK-2 cells were obtained from the American Tissue Culture Collection (ATCC, Rockville, MD, USA). Cells were cultured in DMEM/F12 (Lot. no. 10092016, Gibco, Thermo Fisher Scientific, Waltham, USA) supplemented with 10% FBS (Lot. no. 35081005, Gibco, Thermo Fisher Scientific, Waltham, USA), 100 U/mL penicillin, and 100 μg/mL streptomycin (Lot. no. 127819, Gibco, Thermo Fisher Scientific, Waltham, USA) at 37 °C with 5% CO_2_. Cells were transfected with 50 nM NFAT2 small interfering RNA (siRNA) (No. stB0007239 genOFFTM st-h-NFATC1, RiboBio Co., Ltd. Guangzhou, China) and Lipofectamine 2000 (Lot no. 2125329, Thermo Fisher Scientific, Waltham, USA) for 6 h or were cultured with 100 nM 11R-VIVIT (CAS. No. 592517-80-1, MedChemExpress, New Jersey, USA) for 2 h and then treated with 5 ng/mL [20] TGF-β1 (Lot. AV7321041, R&D Systems, Minneapolis, MN, USA) for 72 h in vitro in serum-free DMEM/F12. A scrambled siRNA (No. siN0000001-1-5 siR NC #1, RiboBio Co., Ltd. Guangzhou, China) was used as a transfection control.

### Western blot analysis

Kidney tissues and HK-2 cells were lysed with RIPA lysis buffer (No. P0013B, Beyotime Institute of Biotechnology, Jiangsu, China) supplemented with the protease inhibitor phenylmethylsulfonyl fluoride (No. P1082, Beyotime Institute of Biotechnology, Jiangsu, China). Lysates were centrifuged for 10 min at 4 °C, and the supernatants were obtained. Nuclear and cytoplasmic proteins were extracted from cells using a Nuclear and Cytoplasmic Protein Extraction Kit (No. KGP1100, Nanjing KeyGen Biotech. Co. Ltd., Jiangsu, China) according to the manufacturer’s instructions. The protein concentrations of the supernatants were determined using a bicinchoninic acid protein assay kit (Lot. no. WF327088, Thermo Fisher Scientific, Waltham, USA). Proteins were subjected to SDS‑PAGE on 7.5%–12% gels and transferred to polyvinylidene difluoride membranes (No. IPVH00010, Millipore, Billerica, MA, USA). The membranes were blocked with 5% nonfat dry milk in Tris-buffered saline-Tween for 1 h at room temperature and incubated overnight at 4 °C with anti-β-actin (1:1,000; cat. no. 8457; Cell Signalling Technology, Danvers, MA, USA), anti-GAPDH (1:5,000; cat. no. AP0063; Bioworld Technology, St. Louis Park, MN, USA), anti-histone H3 (1:1,000; cat. no. AF0863; Affinity Biosciences, USA), anti-NFAT2 (1:1,000; cat. no. ab25916; Abcam, Cambridge, MA, USA), anti-phospho-NFAT2 (Ser 172; 1:1,000; cat. no. AF8293; Affinity Biosciences, USA), anti-fibronectin (1:1,000; cat. no. ab2413; Abcam, Cambridge, MA, USA), anti-α-SMA (1:500; cat. no. ab5694; Abcam, Cambridge, MA, USA), anti-caspase-3 (1:1,000; cat. no. 9662; Cell Signalling Technology, Danvers, MA, USA), anti-cleaved caspase‑3 (1:1,000; cat. no. 9664; Cell Signalling Technology, Danvers, MA, USA), and anti-Bax (1:1,000; cat. no. 2772; Cell Signalling Technology, Danvers, MA, USA) antibodies. The membranes were then incubated with a horseradish peroxidase‑conjugated goat anti‑rabbit (1:3,000; cat. no. 7074; Cell Signalling Technology, Danvers, MA, USA) or anti-mouse (1:3,000; cat. no. 7072; Cell Signalling Technology, Danvers, MA, USA) secondary antibody for 1 h at room temperature. After being washed in TBST, the protein bands were visualized using Pierce™ ECL Western blotting Substrate (Lot: 201209-86, Thermo Fisher Scientific, Waltham, USA) and detected with Image Quant LAS 500 (GE Healthcare Life Sciences, USA). ImageJ software was used to analyse the band intensity, and the data were standardized to β-actin, GAPDH or histone H3 (nuclear fractions).

### mRNA extraction, reverse transcription, and quantitative polymerase chain reaction (RT–qPCR)

Total RNA was extracted from the kidney using TRIzol® reagent (Lot: 326401, Invitrogen, Thermo Fisher Scientific, Waltham, USA), and reverse transcription (RT) was carried out using a PrimeScript RT Reagent Kit (Cat #RR047A, Takara Bio Inc., Madison, WI, USA). cDNA was generated and subjected to PCR using Power SYBR Green PCR Master Mix (Cat #RR820A, Takara Bio Inc., Madison, WI, USA). The data were analysed using the 2^‑ΔΔCq^ method, and GAPDH was used as the internal control. The primer sequences are shown in Table 1.

**Table 1:** Sequences of primers for RT-qPCR.

Gene	Primer sequences
GAPDH (mouse)	Forward: 5ʹ- AGGTCGGTGTGAACGGATTTG-3ʹ Reserve: 5ʹ-TGTAGACCATGTAGTTGAGGTCA-3ʹ
α-SMA (mouse)	Forward: 5ʹ-CCCAGACATCAGGGAGTAATGG-3ʹ Reserve: 5ʹ-TCTATCGGATACTTCAGCGTCA-3ʹ
Fibronectin (mouse)	Forward: 5ʹ-ATGTGGACCCCTCCTGATAGT-3ʹ Reserve: 5ʹ-GCCCAGTGATTTCAGCAAAGG-3ʹ

*RT-qPCR* reverse transcription-quantitative polymerase chain reaction.

### Flow cytometric analysis

A fluorescein isothiocyanate (FITC) annexin-V apoptosis detection kit I was purchased from Dojindo Laboratories (AD10, Kumamoto, Kyushu, Japan) and used to determine the level of apoptosis according to the manufacturer’s instructions. The medium was removed from the cells, and then the cells were washed three times with cold PBS and digested with 0.25% EDTA‑free trypsin (Lot. no. 15050065, Gibco, Thermo Fisher Scientific, Waltham, USA). After centrifugation for 5 min at 140 × *g* at room temperature, the cells were washed twice with cold PBS and resuspended in 1× binding buffer at a concentration of 1 × 10^6^ cells/mL. One hundred microlitres of the solution was transferred to a 5 mL culture tube, and 5 μL of FITC Annexin V and PI were each added. The cells were gently vortexed and incubated for 15 min at room temperature (25 °C) in the dark. Then, 400 μL of 1× binding buffer was added to each tube, and apoptosis was analysed by flow cytometry (CytoFLEX, Beckman Coulter, Indianapolis, USA) within 1 h.

### Immunofluorescence staining

Frozen sections were fixed with 4% paraformaldehyde at room temperature for 15 min, permeabilized with 0.5% Triton X-100 for 10 min, and incubated with 5% BSA for 30 min at room temperature. The frozen sections were incubated with anti-NFAT1 (1:200; cat. no. 5861; Cell Signalling Technology, Danvers, MA, USA), anti-NFAT2 (1:50; cat. no. sc-7294; Santa Cruz Biotechnology, Dallas, TX, USA), anti-NFAT3 (1:100; cat. no. PA1-021; Gibco, Thermo Fisher Scientific, Waltham, USA), and anti-NFAT4 (1:50; cat. no. sc-8405; Santa Cruz Biotechnology, Dallas, TX, USA) antibodies overnight at 4 °C. The frozen sections were washed three times in PBS and incubated with Alexa Fluor 488-conjugated goat anti-rabbit (1:500; cat. no. sc-4412; Santa Cruz Biotechnology, Dallas, TX, USA) or goat anti-mouse (1:500; cat. no. sc-4408; Santa Cruz Biotechnology, Dallas, TX, USA) secondary antibodies for 1 h at room temperature. The sections were counterstained with DAPI to label the nuclei. Images of each frozen section were captured using a confocal microscope (Nikon C2 Confocal Microscope, Nikon), and the mean grey value was analysed using ImageJ software (version 1.52; National Institutes of Health).

### TUNEL staining

TUNEL staining was performed using an in situ Cell Death Detection Kit (LOT 44446200, Roche Diagnostics GmbH, Mannheim, Germany). Frozen sections or cells in 6-well plates were fixed in 4% paraformaldehyde for 20 min at room temperature. After being washed with PBS three times, the frozen sections or cells in 6-well plates were blocked with 3% H_2_O_2_ in methanol for 10 min at room temperature and then permeabilized with 0.1% Triton X‑100 in 0.1% sodium citrate for 10 min at room temperature. Each frozen section or well of cell plates was incubated with 500 μL of TUNEL reaction mixture containing 50 μL of the enzyme solution and 450 μL of the label solution for 60 min at 37 °C in humidified and dark conditions. After being washed three times with PBS, the frozen sections or cells in 6-well plates were stained with DAPI for 10 min at room temperature and placed on coverslips. Six fields were captured for each frozen section (*n* = 3 in each group) using a confocal fluorescence microscope (Nikon C2 Confocal Microscope, Nikon), and positively stained cells were quantified and analysed using ImageJ software (version 1.52; National Institutes of Health).

### Statistical analysis

All data were analysed using SPSS statistical software for Windows, version 23.0 (SPSS, Inc., Chicago, IL, USA). The data were analysed by one-way ANOVA for multiple comparisons, followed by Bonferroni’s *post hoc* analysis. Comparisons between two groups were assessed using Student’s *t* tests. Two-tailed tests were used for all comparisons, and *P* < 0.05 was considered statistically significant.

## Results

### NFAT2 was markedly increased in human renal tissue with severe fibrosis

**Fig. 1:**
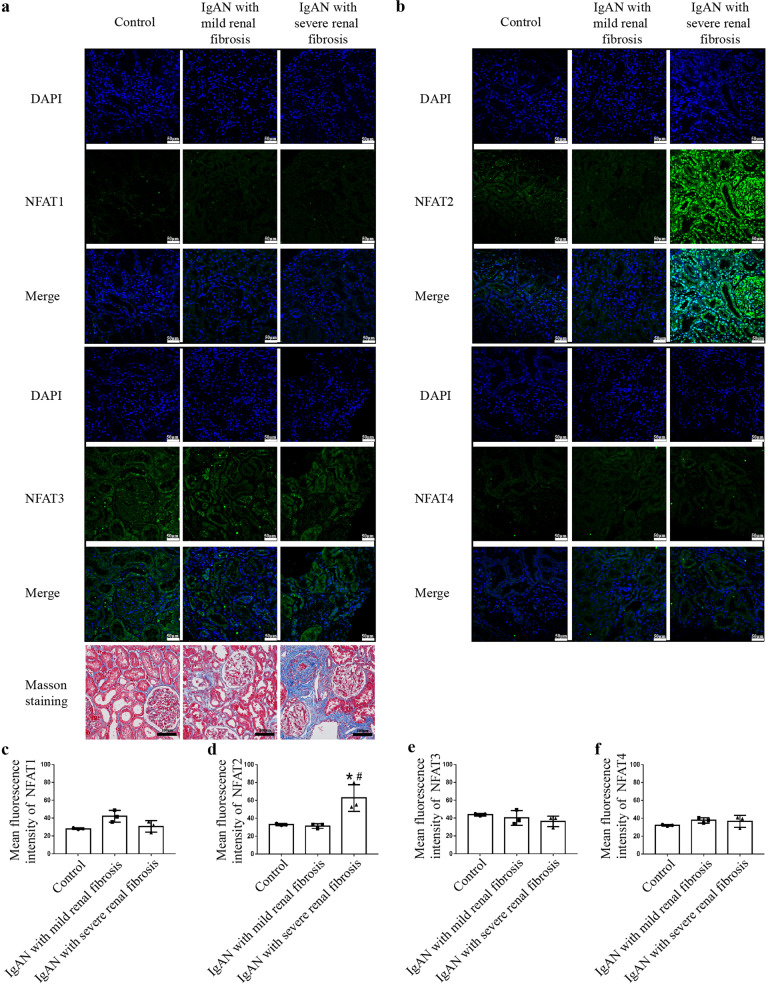
NFAT2 is markedly increased in human renal tissue with severe fibrosis. Immunostaining for NFAT1, NFAT2, NFAT3 and NFAT4 was performed on tissues from IgAN patients and adjacent normal tissues from renal cell carcinoma patients. Representative confocal images showed higher expression and more nuclear localization of NFAT2 in the renal tubules, interstitium and glomerulus in IgAN patients with severe renal fibrosis (*n* = 3) than in IgAN patients with mild renal fibrosis or in adjacent normal renal tissue from renal cell carcinoma patients (*n* = 3) (Scale bars = 50 μm). Masson staining was used to assess kidney fibrosis (scale bars = 100 μm). **a** Immunostaining for NFAT1 and NFAT3. **b** Immunostaining for NFAT2 and NFAT4. **c**–**f** Quantitative analysis for the immunofluorescence of NFAT1, NFAT2, NFAT3 and NFAT4. *P < 0.05 vs. Control; ^#^*P* < 0.05 vs. IgAN with mild renal fibrosis. NFAT1 nuclear factor of activated T cells 1, NFAT2 nuclear factor of activated T cells 2, NFAT3 nuclear factor of activated T cells 3, NFAT4 nuclear factor of activated T cells 4.

It is difficult to obtain human kidney samples during the AKI-to-CKD transition because patients diagnosed with AKI seldom undergo renal biopsies. We evaluated the expression of NFAT1, NFAT2, NFAT3 and NFAT4 in frozen sections from three biopsy-proven IgAN patients with severe renal fibrosis and three IgAN patients with mild renal fibrosis. Adjacent normal renal tissue from three renal cell carcinoma patients was used as a control. Immunofluorescence analysis showed increased the expression and nuclear localization of NFAT2 in the renal tubules, interstitium, and glomerulus in IgAN patients with severe renal fibrosis compared with IgAN patients with mild renal fibrosis and the controls. However, the expression of NFAT1, NFAT3, and NFAT4 was similar in IgAN patients with various degrees of fibrosis and the controls (Fig. 1a–f).

### NFAT2 was activated in the animal model of AKI-to-CKD progression

**Fig. 2:**
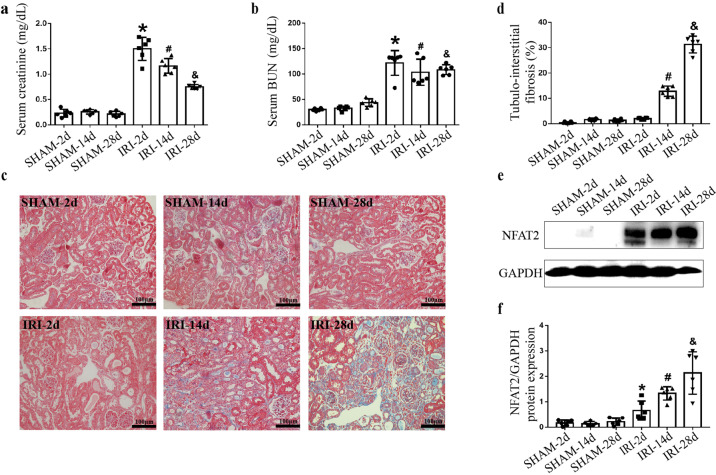
NFAT2 is increased in the renal tissue of AKI-to-CKD transition animal model. **a** Scr and (**b**) serum BUN levels were measured in the sham-operated and IRI models. **c** Masson staining showed renal histopathology fibrosis. Scale bars = 100 μm. **d** Quantification of tubulointerstitial fibrosis. **e**–**f** Protein expression of NFAT2 in the kidneys of sham-operated (2d, 14d, 28d) and IRI (2d, 14d, 28d) mice, as determined by Western blotting and densitometric analysis. *P < 0.05 vs. SHAM-2d, ^#^*P* < 0.05 vs. SHAM-14d, ^&^*P* < 0.05 vs. SHAM-28d. NFAT2 nuclear factor of activated T cells 2, AKI acute kidney injury, CKD chronic kidney disease, Scr serum creatinine, BUN blood urea nitrogen, IRI ischemia-reperfusion injury, NFAT1 nuclear factor of activated T cells 1, NFAT3 nuclear factor of activated T cells 3, NFAT4 nuclear factor of activated T cells 4.

**Fig. 3:**
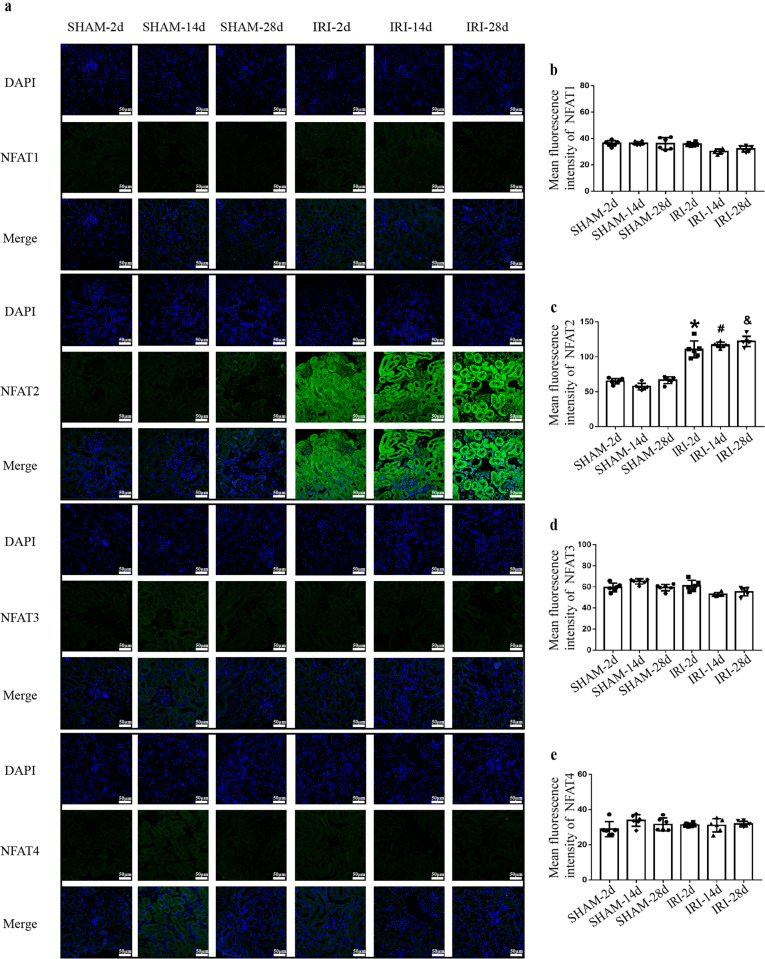
NFAT2 is activated in the RTECs of AKI-to-CKD transition animal model. **a** Immunostaining for NFAT1, NFAT2, NFAT3, NFAT4 (green) and DAPI (blue) was performed in frozen renal sections from sham-operated (2d, 14d, 28d) and IRI (2d, 14d, 28d) mice. Scale bars = 50 μm. **b**–**e** Quantitative analysis for the immunofluorescence of NFAT1, NFAT2, NFAT3 and NFAT4. *P < 0.05 vs. SHAM-2d, ^#^*P* < 0.05 vs. SHAM-14d, ^&^*P* < 0.05 vs. SHAM-28d. NFAT2 nuclear factor of activated T cells 2, RTECs renal tubular epithelial cells, AKI acute kidney injury, CKD chronic kidney disease, NFAT1 nuclear factor of activated T cells 1, NFAT3 nuclear factor of activated T cells 3, NFAT4 nuclear factor of activated T cells 4, IRI ischemia-reperfusion injury.

We used the Bi-IRI model to longitudinally examine CKD progression after AKI. The levels of Scr and BUN were significantly elevated after IRI compared to those of sham-operated mice on the 2nd day. Scr and BUN decreased on the 14th and 28th days compared with the 2nd day but did not completely return to baseline levels after IRI (Fig. 2a and b). Masson staining showed that the degree of renal interstitial fibrosis was more severe on the 14th and 28th days than in sham-operated mice at the corresponding times (Fig. 2c and d). Western blot analysis revealed the higher expression of NFAT2 in the IRI group than in the sham group on the 2nd, 14th, and 28th days, and the expression of NFAT2 in the IRI-28d group was higher than that in the IRI-2d and IRI-14d groups (Fig. 2e and f). Immunofluorescence analysis showed the increased expression and nuclear localization of NFAT2 in RTECs in the IRI-2d, 14d, and 28d groups compared with the corresponding sham-operated groups, while the glomerulus did not show elevated NFAT2 expression. Consistent with our human kidney biopsy results, NFAT1, NFAT3, and NFAT4 were not obviously upregulated in RTECs of the AKI-to-CKD animal model in the IRI-2d, 14d, and 28d groups (Fig. 3a–e). Our results indicated that NFAT2 was increased in RTECs in the AKI-to-CKD progression model.

### 11R-VIVIT attenuated tubulointerstitial fibrosis in the AKI-to-CKD progression model

**Fig. 4:**
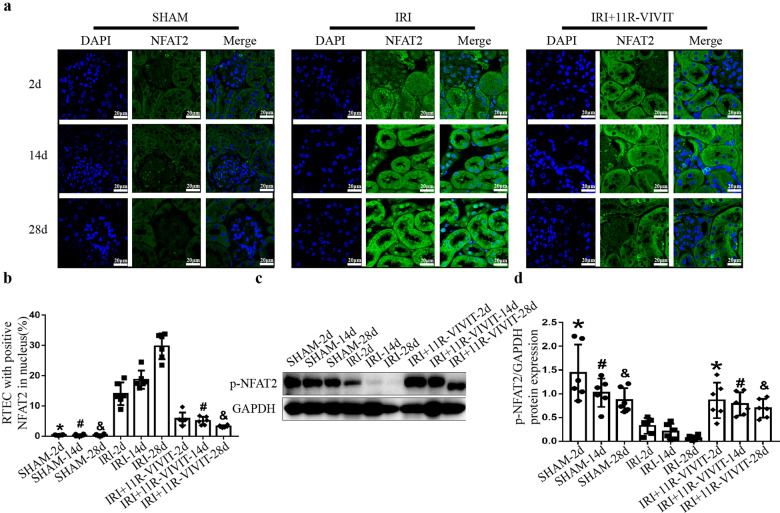
11R-VIVIT inhibits the nuclear localization and dephosphorylation of NFAT2 in RTECs in an AKI-to-CKD progression model. **a** Double immunofluorescence staining of NFAT2 (green) and DAPI (blue) and merged images of kidneys from the sham-operated, IRI and IRI + 11R-VIVIT treatment groups. Scale bars = 20 μm. **b** Quantification of the percentage of renal tubular epithelial cells with NFAT2 expression in the nucleus. *P < 0.05 vs. IRI-2d, ^#^*P* < 0.05 vs. IRI-14d, ^&^*P* < 0.05 vs. IRI-28d. **c** Protein expression of p-NFAT2 (Ser172) in the sham-operated, IRI and IRI + 11R-VIVIT treatment groups on the 2nd, 14th and 28th day. **d** The quantitative results of p-NFAT2 (Ser172) were normalized to GAPDH. NFAT2 nuclear factor of activated T cells 2, RTECs renal tubular epithelial cells, AKI acute kidney injury, CKD chronic kidney disease, IRI ischemia-reperfusion injury, p-NFAT2 phosphorylated nuclear factor of activated T cells 2.

As there is currently no specific NFAT2 inhibitor, we used the NFAT inhibitor 11R-VIVIT to inhibit NFAT2 activity. Mice were treated with 5 mg/kg 11R-VIVIT on Days 1, 3, 10, 17, and 24 after IRI. We examined the inhibitory effect of 11R-VIVIT on the nuclear localization of NFAT2 by immunofluorescence staining. 11R-VIVIT treatment significantly reduced the nuclear localization of NFAT2 in RTECs in the AKI-CKD transition model (Fig. 4a and b). Moreover, Western blot (Fig. 4c and d) analysis showed that the expression of phosphorylated NFAT2 was decreased in the IRI group on the 2nd, 14th, and 28th days and that 11R-VIVIT treatment significantly increased the expression of phosphorylated NFAT2. These results indicated that 11R-VIVIT could inhibit the nuclear localization and dephosphorylation of NFAT2.

**Fig. 5:**
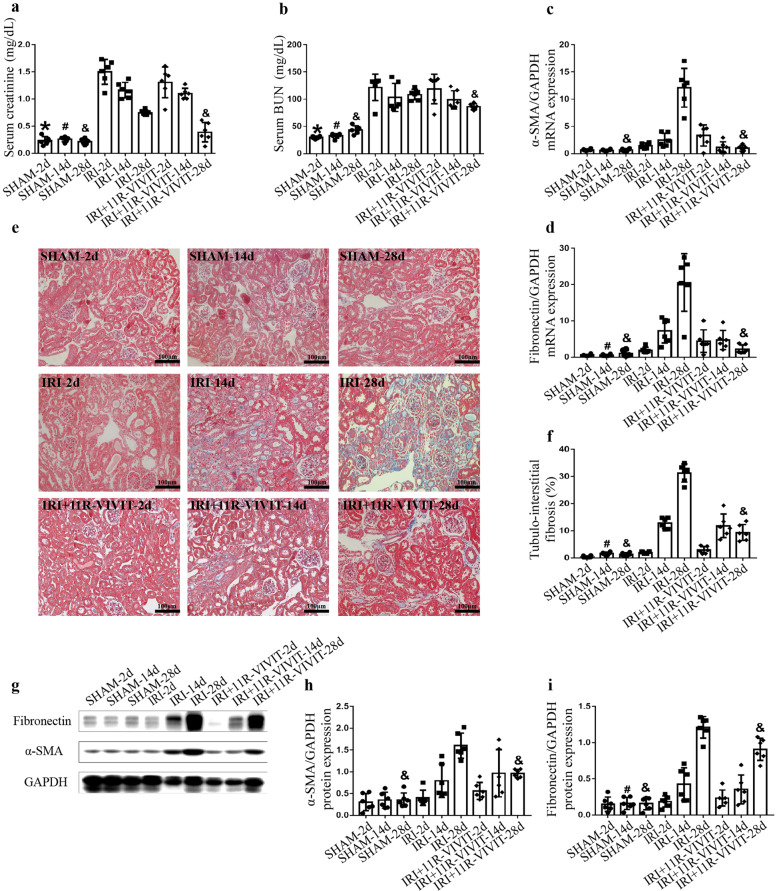
11R-VIVIT attenuates tubulointerstitial fibrosis in an animal model of AKI-to-CKD progression. **a** Scr and (**b**) serum BUN levels were determined in the sham-operated, IRI and IRI + 11R-VIVIT treatment groups on the 2nd, 14th and 28th day. **c**–**d** α-SMA and fibronectin mRNA expression was measured in each group by RT-qPCR on the 2nd, 14th and 28th day. **e** Masson staining showed renal histopathological fibrosis in each group on the 2nd, 14th and 28th day. Scale bars = 100 μm. **f** Quantification of tubulointerstitial fibrosis. **g** Protein expression of α-SMA and fibronectin in each group on the 2nd, 14th and 28th day. **h**–**i** The quantitative results of α-SMA and fibronectin were normalized to GAPDH. *P < 0.05 vs. IRI-2d, ^#^*P* < 0.05 vs. IRI-14d, ^&^*P* < 0.05 vs. IRI-28d. AKI acute kidney injury, CKD chronic kidney disease, Scr serum creatinine, BUN blood urea nitrogen, IRI ischemia-reperfusion injury, RT-qPCR reverse transcription-quantitative polymerase chain reaction.

11R-VIVIT significantly improved renal function compared with that in the IRI group on the 28th day (Fig. 5a and b). 11R-VIVIT treatment also alleviated IRI-induced interstitial fibrosis (Fig. 5e and f). In addition, RT-qPCR (Fig. 5c and d) and Western blot (Fig. 5g, h, and i) analysis revealed that the expression of the fibrotic markers α-SMA and fibronectin was decreased in the IRI + 11R-VIVIT group on the 28th day. These results revealed that 11R-VIVIT could attenuate tubulointerstitial fibrosis in the AKI-to-CKD progression model.

Furthermore, we evaluated the toxic side effects of 11R-VIVIT on other organs, including the heart and liver. HE staining showed that the pathological changes in heart and liver tissue were similar in the sham operation, IRI, and IRI + 11R-VIVIT groups (Supplementary Fig. 1). There was no difference in the level of serum AST between the sham operation, IRI, and IRI + 11R-VIVIT groups (Supplementary Fig. 2). These results indicated that 11R-VIVIT had no toxic side effects on the heart and liver.

### 11R-VIVIT reduced tubular epithelial cell apoptosis in the AKI-to-CKD progression model

**Fig. 6:**
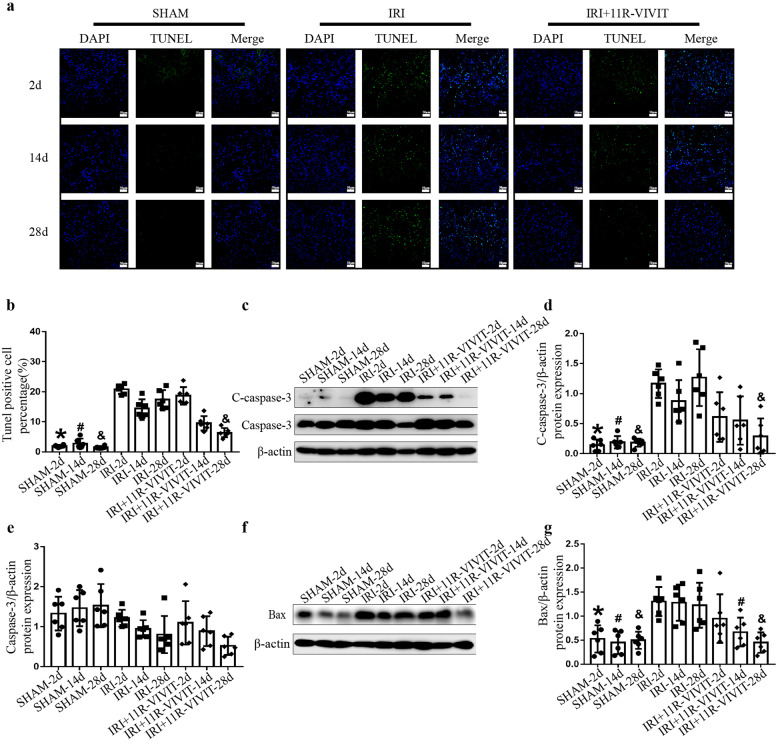
11R-VIVIT attenuates tubular epithelial cell apoptosis in an animal model of AKI-to-CKD progression. **a** TUNEL staining of frozen renal tissue sections from the sham-operated, IRI and IRI + 11R-VIVIT treatment groups on the 2nd, 14th and 28th day. Scale bars = 50 μm. **b** Quantification of TUNEL‑positive tubular epithelial cells in the sham-operated, IRI and IRI + 11R-VIVIT treatment groups on the 2nd, 14th and 28th day. **c** Western blot analysis of the expression of caspase-3 and C‑caspase-3 in each group on the 2nd, 14th and 28th day. **d**–**e** The quantitative results of C-caspase-3 and caspase-3 were normalized to β-actin. **f** Western blot analysis of the expression of Bax in each group on the 2nd, 14th and 28th day. **g** The quantitative results of Bax were normalized to β-actin. *P < 0.05 vs. IRI-2d, ^#^*P* < 0.05 vs. IRI-14d, ^&^*P* < 0.05 vs. IRI-28d. AKI acute kidney injury, CKD chronic kidney disease, RTECs renal tubular epithelial cells, IRI ischemia-reperfusion injury, C‑caspase‑3 cleaved-caspase‑3.

TUNEL staining was used to evaluate apoptosis in the AKI-to-CKD progression animal model. The number of TUNEL-positive RTECs was significantly higher in the IRI group than in the sham-operated group on the 2nd, 14th, and 28th days (Fig. 6a and b). Western blot analysis showed that the expression of cleaved‑caspase-3, a marker of apoptosis, was significantly increased in the kidney tissues of the IRI group on the 2nd, 14th, and 28th days (Fig. 6c, d, and e). Bax, a proapoptotic member of the Bcl‑2 family, was also significantly increased in IRI mice on the 2nd, 14th, and 28th days (Fig. 6f and g). These results indicated that apoptosis was induced in RTECs in the AKI-to-CKD progression model. After 11R-VIVIT intervention, the number of TUNEL‑positive RTECs decreased in the IRI + 11R-VIVIT group compared with the IRI group on the 14th and 28th days (Fig. 6a and b). 11R-VIVIT treatment also reduced cleaved‑caspase-3 (Fig. 6c, d, and e) and Bax (Fig. 6f and g) expression on the 28th day.

### NFAT2-targeted siRNA attenuated TGFβ‑induced tubular epithelial cell apoptosis

**Fig. 7:**
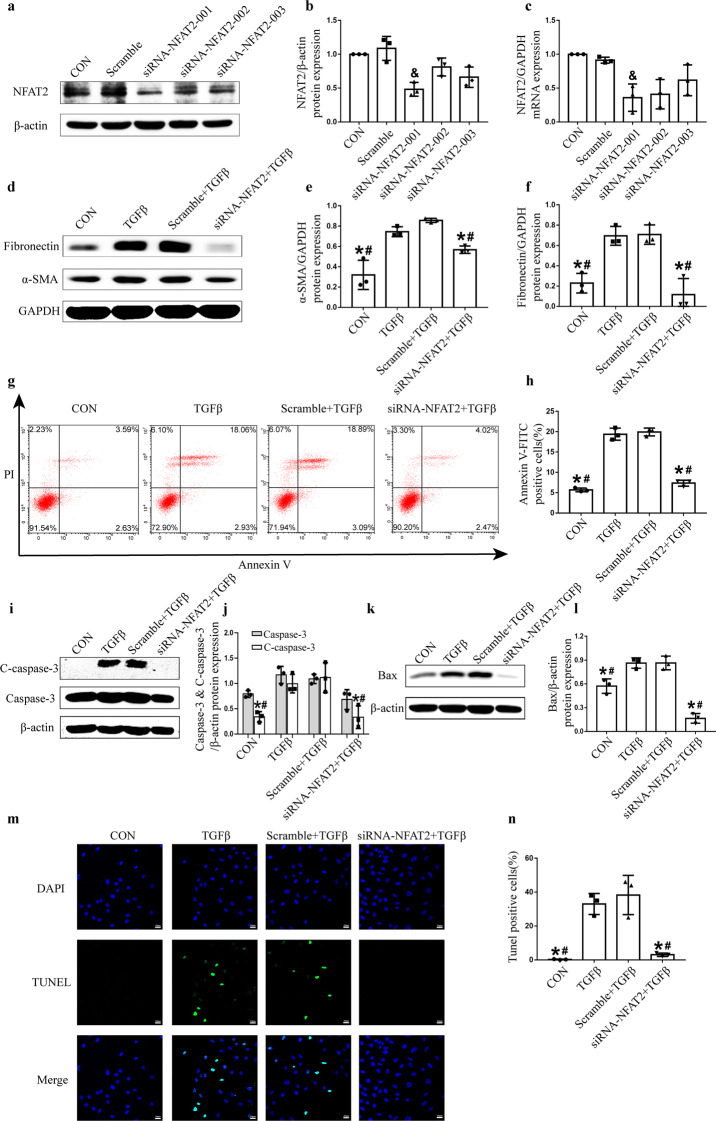
NFAT2-targeted siRNA attenuates RTEC apoptosis in vitro following TGFβ stimulation. HK-2 cells in the TGFβ group were stimulated with 5 ng/mL TGFβ for 72 h. The scramble+TGFβ and siRNA-NFAT2 + TGFβ groups were transfected with 5 nM scramble or siRNA-NFAT2 respectively, prior to TGFβ stimulation. **a**–**c** NFAT2-targeted siRNA significantly reduced the mRNA and protein levels of NFAT2. Three sequences of the NFAT2-targeted siRNA were used in the present study, and sequence 001 induced significant reductions in the protein and mRNA levels of NFAT2. **d** The protein expression of α-SMA and fibronectin in HK-2 cells. **e**–**f** The quantitative results of α-SMA and fibronectin were normalized to GAPDH. **g** Apoptosis was examined by flow cytometry. **h** Quantification of RTEC apoptosis by flow cytometry. **i** Western blot analysis of the expression of caspase-3 and C‑caspase-3 in HK-2 cells. **j** The quantitative results of caspase-3 and C‑caspase-3 were normalized to β-actin. **k** Western blot analysis of the expression of Bax in HK-2 cells. **l** The quantitative results of Bax were normalized to β-actin. **m** TUNEL staining of HK-2 cells in the CON, TGFβ, Scramble+TGFβ and siRNA-NFAT2 + TGFβ group. **n** Quantification of TUNEL‑positive RTECs. Scale bars = 20 μm. ^&^*P* < 0.05 vs. CON and scramble, *P < 0.05 vs. TGFβ, ^#^*P* < 0.05 vs. scramble+ TGFβ. NFAT2 nuclear factor of activated T cells 2, RTECs renal tubular epithelial cells, TGFβ transforming growth factor beta, CON control, C‑caspase‑3 cleaved caspase‑3.

To further investigate the role of NFAT2 in the AKI-to-CKD transition, we used siRNA to inhibit the expression of NFAT2. siRNA targeting the NFAT2-001 sequence significantly reduced the mRNA and protein levels of NFAT2 (Fig. 7a, b, and c). Transfection with NFAT2-targeted siRNA decreased the elevated expression of α-SMA and fibronectin induced by TGFβ treatment (Fig. 7d, e, and f). Moreover, flow cytometry showed that NFAT2-targeted siRNA decreased TGFβ‑induced tubular epithelial cell apoptosis (Fig. 7g and h). Western blot analysis suggested that NFAT2-targeted siRNA decreased the expression of cleaved caspase-3 and Bax in HK-2 cells after TGFβ treatment (Fig. 7i–l). The number of TUNEL‑positive RTECs was significantly increased in HK-2 cells after TGFβ treatment and could be reduced by transfection with NFAT2-targeted siRNA (Fig. 7m and n). These data suggested that NFAT2 could mediate tubular epithelial cell apoptosis.

### 11R-VIVIT attenuated TGFβ‑induced tubular epithelial cell apoptosis in vitro

**Fig. 8:**
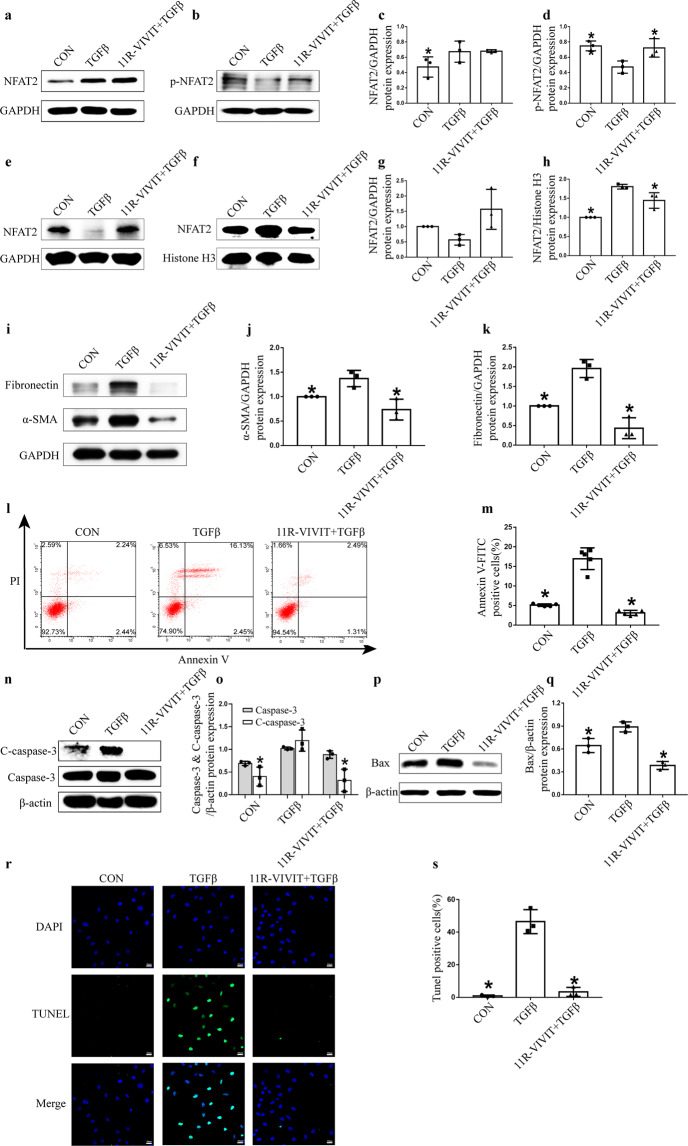
11R-VIVIT attenuates TGFβ‑induced apoptosis in HK-2 cells. **a** TGFβ treatment increased total NFAT2 expression in HK-2 cells. **b** TGFβ treatment decreased p-NFAT2 (Ser172) expression and 11R-VIVIT could increase the protein expression of p-NFAT2 (Ser172). **c** Quantification of NFAT2 expression normalized to GAPDH. **d** Quantification of p-NFAT2 (Ser172) expression normalized to GAPDH. **e**–**f** TGFβ treatment increased nuclear NFAT2 expression in HK-2 cells, and 11R-VIVIT inhibited the nuclear localization of NFAT2. **g** Quantification of NFAT2 expression in the cytoplasmic fraction HK-2 cells; The results were normalized to GAPDH. **h** Quantification of NFAT2 expression in the nuclear fraction of HK-2 cells; The results were normalized to Histone H3. **i** The protein expression of α-SMA and fibronectin in HK-2 cells. **j**–**k** The quantitative results of α-SMA and fibronectin were normalized to GAPDH. **l** Apoptosis was examined by flow cytometry. **m** Quantification of RTEC apoptosis by flow cytometry. **n** Western blot analysis of the expression of caspase-3 and C‑caspase-3 in HK-2 cells. **o** The quantitative results of caspase-3 and C‑caspase-3 were normalized to β-actin. **p** Western blot analysis of the expression of Bax in HK-2 cells. **q** The quantitative results of Bax were normalized to β-actin. **r** TUNEL staining of HK-2 cells in the CON, TGFβ and 11R-VIVIT + TGFβ group. **s** Quantification of TUNEL‑positive tubular epithelial cells. Scale bars = 20 μm. *P < 0.05 vs. TGFβ. TGFβ transforming growth factor beta, NFAT2 nuclear factor of activated T cells 2, p-NFAT2 phosphorylated nuclear factor of activated T cells 2, RTECs renal tubular epithelial cells, C‑caspase‑3 cleaved caspase‑3.

The effect of 11R-VIVIT on NFAT2 activity in HK-2 cells after TGFβ treatment was assessed in vitro. TGFβ treatment increased the expression of NFAT2 (Fig. 8a, c). Moreover, it decreased the expression of phosphorylated NFAT2 (Fig. 8b, d) and increased the nuclear localization of NFAT2 in HK-2 cells, and these effects were blocked by the administration of 11R-VIVIT (Fig. 8e–h). 11R-VIVIT also decreased the elevated expression of α-SMA and fibronectin induced by TGFβ treatment (Fig. 8i, j, and k). Flow cytometry showed that 11R-VIVIT decreased TGFβ‑induced tubular epithelial cell apoptosis (Fig. 8l and m). 11R-VIVIT also decreased cleaved caspase-3 and Bax levels in HK-2 cells after TGFβ treatment (Fig. 8n–q). The increased number of TUNEL‑positive RTECs induced by TGFβ treatment was reduced by 11R-VIVIT treatment (Fig. 8r and s). These data suggested that 11R-VIVIT could inhibit TGF-β-induced tubular epithelial cell apoptosis in vitro.

## Discussion

In this study, we demonstrated that, among the NFAT subtypes, only the expression of NFAT2 varies with the degree of fibrosis. NFAT2 was markedly increased in IgAN patients with severe renal fibrosis, while the expression of NFAT1, NFAT3, and NFAT4 was similar in IgAN patients with various degrees of fibrosis and the controls. As renal fibrosis is a common consequence of various progressive kidney diseases, including the AKI-to-CKD transition, we further explored the expression of NFAT subtypes in the animal model of AKI-to-CKD progression. Consistently, we found that NFAT2 but not the other NFAT subtypes was significantly upregulated in RTECs, accompanied by increased nuclear accumulation of NFAT2. These data indicated that NFAT2 in RTECs was activated in AKI-to-CKD progression.

Increasing evidence has demonstrated that NFAT2 plays a proapoptotic role through the intrinsic and extrinsic apoptotic pathways [23–25]. A previous study [23] showed that the activation of NFAT2 in cardiomyocytes could induce apoptosis through the NFAT2/Fas/FasL signalling pathway. Similarly, activated NFAT2, as a transcription factor, upregulated FasL expression and induced apoptosis in mouse Leydig tumour cells [24]. In addition, another study demonstrated that colorectal cancer cell apoptosis could be induced through the NFAT2/p53 pathway [18], which could activate the mitochondrial-mediated endogenous apoptotic pathway in response to genotoxic and environmental stress. Our previous studies have also shown that NFAT2 mediates high-glucose-induced glomerular podocyte apoptosis through increased Bax expression [15]. As an important mechanism of AKI-to-CKD progression, excessive apoptosis in RTECs could lead to inflammatory cell infiltration and cytokine secretion, further promoting renal fibrosis. Inhibiting tubular epithelial cell apoptosis could ameliorate renal fibrosis [9, 10, 25]. In our study, we demonstrated that RTEC apoptosis was significantly increased in an animal model of AKI-to-CKD transition. In vitro experiments also showed that inhibiting NFAT2 could ameliorate the HK-2 cell apoptosis induced by TGFβ. Therefore, we hypothesized that NFAT2 may induce renal fibrosis in AKI-to-CKD progression through the induction of apoptosis in RTECs.

However, one recent study indicated that NFAT2 played a renoprotective role by promoting epithelial regeneration after AKI induced by mercuric chloride, and NFAT2-knockout mice exhibited increased apoptosis, sustained injury, and delayed regeneration [13], which contradicted the proapoptotic role of NFAT2 in renal fibrosis in our study. As different mechanisms are involved in AKI and renal fibrosis, the same signalling pathways or pathogenesis may play different roles in these two processes. For example, autophagy is induced in RTECs during AKI and protects against kidney injury [26]. However, during the AKI-to-CKD transition, the sustained activation of autophagy leads to maladaptive responses and promotes renal fibrosis [27, 28]. Therefore, inhibiting NFAT2 might result in different effects on RTEC apoptosis in AKI and AKI-to-CKD transition. Moreover, it is well known that the mechanisms of kidney injury induced by IRI or toxicants are distinct. In our study, we proved that IR-AKI induced the sustained activation of NFAT2 in RTECs at 28 days. However, in AKI induced by mercuric chloride, NFAT2 expression was significantly increased at Day 3 but returned to baseline levels at Day 5, indicating that different mechanisms associated with NFAT2 are involved in different AKI models. In addition, although NFAT2 attenuation resulted in increased RTEC apoptosis in AKI induced by mercuric chloride, there was no difference in the kidney injury score or Scr levels between wild-type and NFAT2-knockout mice.

Based on the above research results, we hypothesized that inhibiting NFAT2 may be a potential therapeutic strategy for preventing renal fibrosis after AKI. CaN dephosphorylates multiple serine residues near the N-termini of NFAT proteins, enabling them to translocate from the cytoplasm to the nucleus. Traditional CNIs, including cyclosporine and tacrolimus, inhibit CaN phosphatase activity and further restrain the activation of NFAT [29]. However, the application of CNIs is associated with the progressive loss of renal function, hypertension, neurotoxicity, and an increased risk of malignancy. Intrarenal expression of TGFβ and other profibrogenic molecules was significantly increased in patients with tacrolimus and cyclosporine nephrotoxicity, which further promoted tubulointerstitial fibrosis [30]. Moreover, other intracellular profibrogenic signalling pathways, including mitogen-activated protein kinase (MAPK) and extracellular signal-regulated kinase (ERK), were also activated by CNI treatment [31, 32], which restricts the use of CNIs in the AKI-to-CKD transition.

11R-VIVIT is a membrane-permeable NFAT inhibitory peptide composed of several consecutive arginines. 11R-VIVIT was developed based on the conserved CaN docking site within NFAT family members. The 11R-VIVIT peptide interferes selectively with the CaN-NFAT interaction without affecting CaN phosphatase activity. Compared with CNIs, 11R-VIVIT has a relatively specific inhibitory effect on the nuclear localization of NFAT2 [21]. In our study, we found that 11R-VIVIT could effectively inhibit the nuclear localization of NFAT2 in animal experiments and in vitro. Moreover, our findings showed that 11R-VIVIT could attenuate renal fibrosis and improve renal function after IR-AKI. We also found that 11R-VIVIT treatment could effectively attenuate RTEC apoptosis in vitro and in vivo. These data indicated that 11R-VIVIT has a protective effect on the AKI-to-CKD transition. Recent basic research has also revealed its value in tissue repair and the treatment of inflammatory diseases, including fracture healing [33], hearing loss [34], autoimmune encephalomyelitis [35], and cardiovascular disorders [36]. Therefore, as a small peptide, 11R-VIVIT has promising future clinical applications.

The possible mechanism of the increased nuclear translocation of NFAT2 in the AKI-to-CKD transition remains unclear. Recent research has indicated that TRPC6 mediates renal fibrosis and that TRPC6 knockout ameliorates kidney fibrosis [37]. Moreover, the inhibition of TRPC6 suppressed NFAT2 nuclear translocation [38]. Therefore, we speculated that the nuclear translocation of NFAT2 may be partly mediated by TRPC6 in the AKI-to-CKD transition.

There are some limitations of this study. We did not specifically attenuate NFAT2 in RTECs in an animal model of AKI-to-CKD transition. In future studies, RTEC-inducible NFAT2-knockout mice will be used to further determine the role of NFAT2 in the AKI-to-CKD transition.

In summary, we demonstrated that NFAT2 was activated in renal fibrosis during AKI-to-CKD progression, and the cell-permeable NFAT inhibitor 11R-VIVIT could effectively inhibit NFAT2 activation, reduce RTEC apoptosis, and prevent AKI-to-CKD progression.

## Supplementary information

Supplementary Figure 1

Supplementary Figure 2

Supplementary Figure 3

Supplementary Table 1
