# Supplementary figures and images for: NFAT inhibitor 11R-VIVIT ameliorates mouse renal fibrosis after ischemia-reperfusion-induced acute kidney injury
Source: Acta Pharmacol Sin. 2021 Dec 22;43(8):2081–93. doi: 10.1038/s41401-021-00833-y (PMC9343462; doi:10.1038/s41401-021-00833-y)

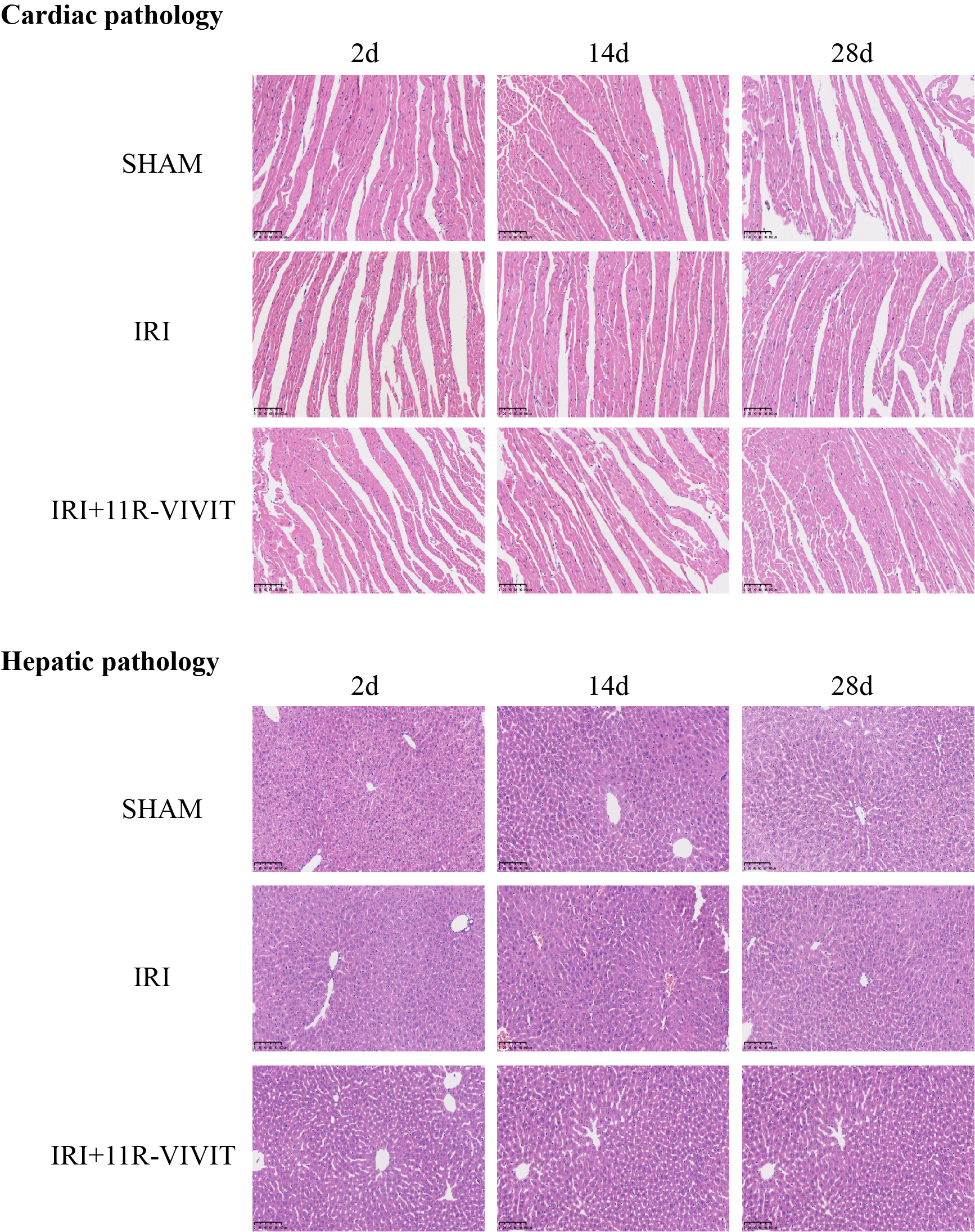

Supplement: Supplementary file 1 — Supplementary Figure 1 [file 41401_2021_833_MOESM1_ESM.jpg]

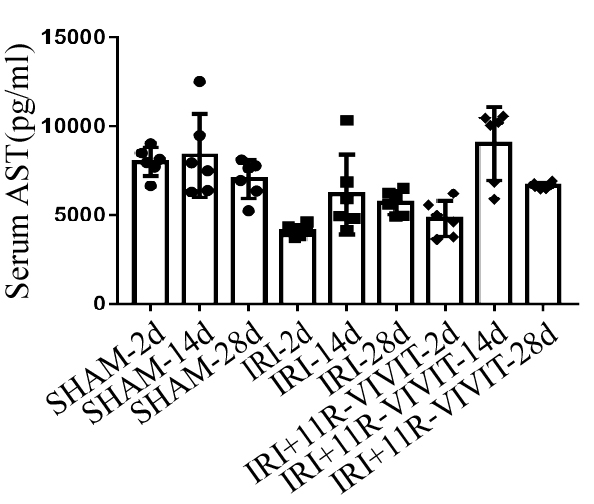

Supplement: Supplementary file 2 — Supplementary Figure 2 [file 41401_2021_833_MOESM2_ESM.jpg]

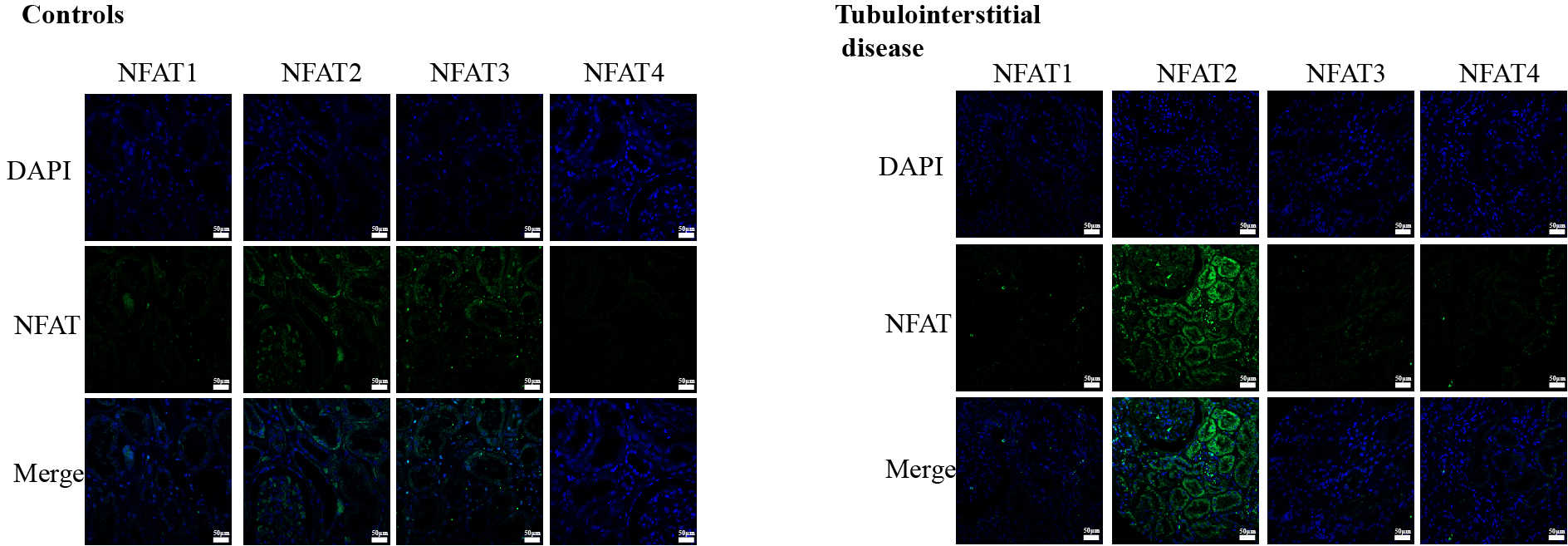

Supplement: Supplementary file 3 — Supplementary Figure 3 [file 41401_2021_833_MOESM3_ESM.jpg]
