# Supplementary material for: NFAT inhibitor 11R-VIVIT ameliorates mouse renal fibrosis after ischemia-reperfusion-induced acute kidney injury
Source: Acta Pharmacol Sin. 2021 Dec 22;43(8):2081–93. doi: 10.1038/s41401-021-00833-y (PMC9343462; doi:10.1038/s41401-021-00833-y)
Supplement: Supplementary file 4 — Supplementary Table 1 [file 41401_2021_833_MOESM4_ESM.docx]

**Supplementary table 1. The clinical parameters of patients performed with immunofluorescence.**

| Patients ID | | Pathology | Age | | Gender | Scr  (μmol/L) | uPCR  (mg/g Cr) | uACR  (mg/g Cr) | ALB  (g/L) | HGB  (g/L) | Uric acid  (μmol/L) | MESTC  score |
| --- | --- | --- | --- | --- | --- | --- | --- | --- | --- | --- | --- | --- |
| 1 | IgAN with severe renal fibrosis | | 50 | Female | 85.4 | 1765 | 1170 | 39.3 | 97 | 628.9 | M1E1S1T1C1 |  |
| 2 |  |  | 58 | Female | 107.2 | 653 | 451 | 42.3 | 117 | 399.1 | M1E0S0T1C1 |  |
| 3 |  |  | 42 | Male | 166.3 | 2697 | 1993 | 40.7 | 115 | 467.9 | M1E0S1T2C0 |  |
| 4 | IgAN with mild renal fibrosis | | 40 | Female | 69.2 | 524 | 311 | 45.8 | 109 | 247.5 | M1E0S0T0C1 |  |
| 5 |  |  | 41 | Female | 60.9 | 695 | 467 | 39.8 | 129 | 355.2 | M1E0S1T0C1 |  |
| 6 |  |  | 26 | Female | 72.3 | 1789 | 1556 | 40.3 | 143 | 256.1 | M1E0S0T0C1 |  |
| 7 | Renal cell carcinoma patients | | 71 | Male | 79.9 | - | - | 34.1 | 125 | 634.3 | --- |  |
| 8 |  |  | 48 | Female | 65.1 | - | - | 45.9 | 78 | 209.0 | --- |  |
| 9 |  |  | 56 | Female | 48.4 | - | - | 45.6 | 140 | 318.9 | --- |  |

Scr, serum creatinine; uPCR, urine protein creatinine ratio; uACR, urinary albumin creatinine ratio; ALB, serum albumin; HGB, hemoglobin; MESTC, mesangial hypercellularity (M), endocapillary cellularity(E), segmental sclerosis (S), interstitialfibrosis/tubular atrophy (T) and crescents(C)
